# Functional analysis of the HD-Zip transcription factor genes *Oshox12* and *Oshox14* in rice

**DOI:** 10.1371/journal.pone.0199248

**Published:** 2018-07-20

**Authors:** Jingxia Shao, Imran Haider, Lizhong Xiong, Xiaoyi Zhu, Rana Muhammad Fraz Hussain, Elin Övernäs, Annemarie H. Meijer, Gaisheng Zhang, Mei Wang, Harro J. Bouwmeester, Pieter B. F. Ouwerkerk

**Affiliations:** 1 College of Life Sciences, Northwest A&F University, Shaanxi, People’s Republic of China; 2 Institute of Biology (IBL), Leiden University, Leiden, The Netherlands; 3 Laboratory of Plant Physiology, Wageningen University and Research Centre, Wageningen, The Netherlands; 4 National Key Laboratory of Crop Genetic Improvement, National Center of Plant Gene Research (Wuhan), Huazhong Agricultural University, Wuhan, People’s Republic of China; 5 Key Laboratory of Biology and Genetic Improvement of Oil Crops of Ministry of Agriculture, Oil Crops Research Institute of the Chinese Academy of Agricultural Sciences, Wuhan, People’s Republic of China; 6 Department of Physiological Botany, EBC, Uppsala University, Uppsala, Sweden; 7 College of Agronomy, Northwest A&F University, Shaanxi, People’s Republic of China; 8 Leiden University European Center for Chinese Medicine and Natural Compounds, Leiden, The Netherlands; National Taiwan University, TAIWAN

## Abstract

The homeodomain-leucine zipper (HD-Zip) transcription factor family plays vital roles in plant development and morphogenesis as well as responses to biotic and abiotic stresses. In barley, a recessive mutation in *Vrs1* (*HvHox1*) changes two-rowed barley to six-rowed barley, which improves yield considerably. The *Vrs1* gene encodes an HD-Zip subfamily I transcription factor. Phylogenetic analysis has shown that the rice HD-Zip I genes *Oshox12* and *Oshox14* are the closest homologues of *Vrs1*. Here, we show that *Oshox12* and *Oshox14* are ubiquitously expressed with higher levels in developing panicles. Trans-activation assays in yeast and rice protoplasts demonstrated that Oshox12 and Oshox14 can bind to a specific DNA sequence, AH1 (CAAT(A/T)ATTG), and activate reporter gene expression. Overexpression of *Oshox12* and *Oshox14* in rice resulted in reduced panicle length and a dwarf phenotype. In addition, *Oshox14* overexpression lines showed a deficiency in panicle exsertion. Our findings suggest that Oshox12 and Oshox14 may be involved in the regulation of panicle development. This study provides a significant advancement in understanding the functions of HD-Zip transcription factors in rice.

## Introduction

Plant genomes contain a large number of transcription factors (TFs) that regulate the expression of several downstream targets. In *Arabidopsis*, approximately 1,500 TFs have been identified and are divided into a number of classes, such as the MADS box, AP2/ERF, Dof, Myb, Hsp, bZIP, NAC and homeobox genes [1–3]. In addition, the rice genome contains more than 1,600 TFs, accounting for 2.6% of its estimated 56,797 genes [4]. Homeobox (HB) TFs belong to a large gene family characterized by the presence of a conserved 61 amino acid sequence known as the homeodomain (HD) motif which is responsible for sequence-specific DNA binding. Of these HB TFs, roughly half are so-called homeodomain-leucine zipper (HD-Zip) proteins, which also contain a leucine zipper motif [5]. The HD-Zip proteins are unique to plants and do not occur in other eukaryotes [6, 7–9]. To date, 48 and 47 HD-Zip members have been found in *Arabidopsis* and rice, respectively [7, 10, 11–14]. The HD-Zip proteins have been classified into four subfamilies (HD-Zip I to IV) on the basis of sequence similarities and the exon/intron patterns of the genes [11, 12, 15]. The roles of the HD-Zip TFs have been determined largely through work in *Arabidopsis* and rice, and these roles are associated with various biological functions, including vascular development, leaf polarity, embryogenesis, meristem regulation and developmental responses to environmental conditions [10, 14, 16–18].

Especially, among the HD-Zip I family, many members in several plant species are involved in developmental regulation in response to changes in environmental conditions [17]. For example, *Arabidopsis Athb-5*, -*6*, -*7*, and -*12* [11, 19, 20], sunflower *Hahb4* [21, 22], Medicago *Mthb1* [23], tobacco *Nahd20* [24] and maize *Zmhdz-1* and *-10* [25, 26], are mainly induced by water deficit, salt and abscisic acid (ABA). Furthermore, in rice *Oshox6*, -*22* and -*24*, the closest homologues of *Athb-7* and -*12*, are also upregulated by water deficit [7, 12, 27, 28] while *Oshox4* is downregulated under drought conditions [12] and also plays a role in gibberellin (GA) signaling [12, 29]. Several reports have shown the functions of HD-Zip I genes in developmental processes. In tomato, *LeHB1*, is highly expressed in flowers and developing fruits, and its overexpression altered floral organ morphology of [30]. In *Arabidopsis*, the abiotic stress-responsive gene *Athb-12* was recently also found to regulate leaf growth by promoting cell expansion and endoreduplication [31]. In cucumber, *Cucumis sativus Glabrous 1* (*CsGL1*) encodes an HD-Zip I protein. In addition, *CsGL1* is also strongly expressed in trichomes and fruit spines and has been shown to be required for trichome formation [32, 33].

In barley, the *Vrs1* (*HvHox1*) gene is encoded by an HD-Zip I underlying a major QTL for grain number, and it determines the difference between two-rowed and six-rowed spikes [34, 35]. The temporal and spatial specificity of *Vrs1* expression indicates that *Vrs1* is involved in the development of lateral spikelets in two-rowed barley. Loss of function in *Vrs1* results in complete conversion of the rudimentary lateral spikelets in two-rowed barley into fully developed fertile spikelets in the six-rowed phenotype [34, 36]. So far, *Vrs1* is the only HD-Zip I gene that has been directly connected to a major yield QTL. In rice, grain yield is mainly determined by three traits: grain weight, number of grains per panicle, and number of panicles. From the viewpoint of increasing rice yield, increasing the grain number per panicle is the main approach to obtaining high yield, and thus, characterizing the rice *Vrs1* homologs, *Oshox12* and *Oshox14*, is of considerable interest. Here, we report a functional analysis of the HD-Zip I genes *Oshox12* and *Oshox14* in rice. We analyzed their transactivation properties, identified novel interaction partners and established their nuclear localization. In addition, we show that *Oshox12* and *Oshox14* may be involved in the regulation of panicle development in rice. Therefore, the present study contributes to a molecular understanding that will support future improvements in grain yield in rice.

## Materials and methods

### Phylogenetic analysis

Alignment of full-length amino acid sequences was performed with ClustalW2 software (http://www.ebi.ac.uk/Tools/clustalw2/). The neighbour-joining method and Poisson correction model were used for phylogenetic tree construction in MEGA version 4.0 [37].

### Binary vector construction

The construct Pro35S-Oshox12 was derived by transferring the full length *Oshox12* (LOC_Os03g10210, MSU Osa1 Release 7) cDNA clone from λFLC-1-B-Oshox12 (GenBank accession AK073446) as a *Kpn*I-*Eco*RI fragment to expression vector pC1300intB-35SnosBK (GenBank accession AY560326). This binary vector allows expression the Cauliflower Mosaic Virus (CaMV) 35S promoter and has a *nos* transcription termination signal. For construct Pro35S-Oshox14, the full-length *Oshox14* (LOC_Os07g39320, MSU Osa1 Release 7) cDNA was cut from λFLC-1-B-Oshox14 (GenBank accession AK121889) with *Bam*HI and *Eco*RI, and then subcloned between *Bgl*II and *Eco*RI of vector pC1300intB-35SnosBK.

For the *Oshox12* promoter-GUS fusion, a 2,869 bp DNA sequence upstream of the predicted translation start site was amplified by PCR from genomic Nipponbare DNA using Phusion polymerase. The primers ProOshox12Fw (5’-CGATCGGATCCATAAGAAACACCTC-3’) and ProOshox12Rev (5’-CTCACGGCCCATGGTCCGAGCGAAC-3’) with *Bam*HI and *Nco*I sites, respectively, were used. This fragment was subsequently cloned into pCAMBIA-1391Z (GenBank accession AF234312) for translational fusion to the β-glucuronidase (GUS) gene, resulting in construct ProOshox12-GUS. With the same strategy, a 2,623 bp PCR product was inserted into pCAMBIA-1391Z, resulting in construct ProOshox14-GUS, except that the primers used were ProOshox14Fw (5’- CTGCTGATAGTGGGATCCACTCTCGGCAAC-3’) and ProOshox14Rev (5’- TCCATGGCGTCTCGCACACTAGCTCGAT-3’).

### Plant transformation and growth conditions

*Oryza sativa* (L.) *Japonica* cultivar Zhonghua 11 was used for stable rice transformation. Embryonic calli were induced on scutella from germinated seeds and rice transformation with the binary vector constructs was performed as described previously except that *Agrobacterium tumefaciens* strain *LBA4404* was used [38]. Prior to growth in the greenhouse, transgenic seedlings were selected on a half-strength Murashige-Skoog medium supplied with 0.7% type I agarose (Sigma) and 25 mg/mL hygromycin B (Duchefa, Haarlem, The Netherlands). Regenerated transgenic plantlets were transferred to the greenhouse and grown in hydroponic culture with a regime of 16 h light, 28°C and 85% relative humidity.

Transgenic and wild type rice seeds were first surface sterilized with 70% ethanol for 30 s and 2% sodium hypochlorite (v/v) for 30 min. The seeds were then rinsed five times in sterile water and immersed in water in the dark for two days at 28°C to induce germination. Additionally, transgenic seeds were selected for one week on half-strength MS media containing hygromycin B to screen transgenic plants. Finally, the germinated seeds were transferred to the greenhouse in three L pots (diameter 19 cm, depth 14.5 cm) filled with soil. The conditions in the greenhouse were as follows: temperature, 28°C day/25°C night; photoperiod, 12 -h light/dark; 85% relative humidity, and 450 μM m^−2^ s^−1^ light intensity. Plants were watered twice a week using modified half-strength Hoagland nutrient solution [39].

To evaluate the agronomic traits of the transgenic rice plants, plant height, number of tillers per plant, panicle length and number of primary branches per panicle, were measured at maturity in ten plants from each of three independent lines. The data were analyzed by Student’s *t*-test. The plant height was measured from the base of the stem to the top of the flag leaf.

### Yeast one-hybrid system

To study the DNA binding properties of the Oshox12 and Oshox14 proteins, expression vectors for use in the yeast one-hybrid system were made. The full length cDNA of *Oshox12* was amplified from λFLC-1-B-Oshox12 with the Oshox12 cDNAFW (5’-CGGAATTCCCATGGGCCGTGAGGAGGATGAGAAG-3’) and Oshox12/14 cDNAREV 5’-(GCGTCGACCCCTCGACGGATCAGGCCCTTA-3’) primers, then the *Eco*RI and *Sal*I fragment of the *Oshox12* full length open reading frame (ORF) was cloned into yeast expression vector pRED-ATGb cut with the same restriction enzyme, resulting in pRED-ATGb-Oshox12.

For the yeast expression vector of *Oshox14*, the full length cDNA of *Oshox14* was first amplified from λFLC-1-B-Oshox14 with the *Oshox14* cDNAFW (5’-CGGAATTCCCATGGACCGATACGGCGAGAAGCA-3’) and Oshox12/14 cDNAREV primers, and then the *Nco*I and *Xho*I fragment of *Oshox14* ORF was cloned into pACTII (pACTII-Oshox14). After the sequence was confirmed, the *Nco*I and *Bgl*II fragment of Oshox14 derived from pACTII-Oshox14 was subcloned into pUC28 (pUC28-Oshox14) and cut with *Nco*I and *Bam*HI. The *Eco*RI and *Sal*I fragment of the *Oshox14* full-length ORF from pUC28-Oshox14 was then inserted into pRED-ATGa with the same enzymes in frame, resulting in pRED-ATGa-Oshox14. Yeast transformations were performed by the LiAc method, essentially as described by Gietz [40]. Yeast transformants were grown on a selective medium without histidine and uracil but with 10 mM 3-AT (to suppress background growth on CM minimal medium lacking histidine) [41]. The yeast reporter strains 4AH1-HIS3 and 4AH2-HIS3 have been described previously [42, 43]. These strains contain tetramers of the AH1 (CAAT(A/T)ATTG) and AH2 (CAAT(C/G)ATTG) sequences which are consensus binding sites for HD-Zip I and II proteins respectively. The 4AH1 and 4AH2 sequences are in front of the *HIS3* reporter gene which is integrated via the pINT1 yeast one-hybrid system at the non-essential PDC6 locus [42]. All handlings of yeast were performed as described previously [43–45].

### Protoplast isolation and transformations

Protoplast isolation was performed as described by Chen [46]. To isolate protoplasts from young seedling tissues, rice seeds were germinated on half-strength MS medium under light for three days. Seedlings were then cultured on the same medium in the dark at 28°C for 10–12 days. Tissues of young seedlings (the stems including sheaths) were cut into approximately 0.5 mm strips and placed in a dish containing K3 medium [47] supplemented with 0.4 M sucrose, 1.5% cellulase R-10 (Yakult Honsa) and 0.3% macerozyme R-10 (Yakult Honsha). The chopped tissue was vacuum-infiltrated and digested at 28°C with gentle shaking at 40 rpm. After incubation, the K3 enzyme medium was replaced by the same volume of W5 solution (154 mM NaCl, 125 mM CaCl_2_, 5 mM KCl and 2 mM MES, adjusted to pH 5.8 with KOH). Protoplasts were released by shaking at 40 rpm for 1 h, followed by filtering through a 35 μm nylon mesh. Protoplasts were collected by centrifuging at 1,300 g for 5 min at 4°C. Pellets were resuspended in suspension solution (0.4 M mannitol, 20 mM CaCl_2_, 5 mM MES, adjusted to pH 5.8 with KOH). Transfection with effector/reporter constructs was performed as follows: 200 μL (usually 1.5~2.5×10^6^ cells/mL) of suspended protoplasts was added to the tube with 10 μg plasmid DNA (including the effector and reporter); then, 220 μL of 40% (w/v) PEG 4,000 prepared with 0.1 M Ca(NO_3_)_2_ and 0.4 M mannitol solution, pH 7, was added, and the mixture was incubated at room temperature for 20 min. After incubation, 750 μL W5 medium was added slowly without mixing, and the protoplasts were transferred to a microtiter plate (12 wells) with 750 μL W5 medium, which was incubated overnight in a room at 25°C in the dark [46]. After 16 h incubation, protoplasts were harvested and lysed in GUS extraction buffer. After centrifugation, the soluble protein concentration was determined using the Bradford assay [48].

To make the effector constructs pRT101-Oshox12 and pRT101-Oshox14, the full length cDNAs of *Oshox12* and *Oshox14* were cut as *Eco*RI-*Bam*HI fragments from λFLC-1-B-Oshox12 and λFLC-1-B-Oshox14 respectively, and cloned into pRT101 cut with the same restriction enzymes [49].

### Subcellular localization analysis

To prepare the GFP-tagged translational fusion constructs, the coding region of *Oshox12* was amplified by PCR from construct Pro35S-Oshox12 using primers 35Sfor1 (5’-ATCCCACTATCCTTCGCAAGACCC-3’) and Oshox12GFPR (5’-CATGCCATGGCGCTGAATTGGTCGTAGA-3’). *Oshox14* was amplified by PCR from construct Pro35S-Oshox14 with primers 35Sfor1 and Oshox14GFPR (5’-CATGCCATGGCGATCAATCCATACAGG-3’). The resulting fragments were cut with *Sal*I and *Nco*I and fused in frame to the N-terminus of the sGFP (S65T) coding sequence under the control of the CaMV 35S promoter in vector pTH-2 [50] and the sequences were confirmed (Baseclear, The Netherlands). Subcellular localization of the *Oshox12-GFP* and *Oshox14-GFP* fusion proteins and a *GFP* control in protoplast using transient transformation was performed as described above. The GFP signal was visualized with confocal laser scanning microscopy (Leica SP5) at 16 h after transformation.

### Southern and northern blot hybridization

Southern and northern blot analyses were performed as described by Memelink et al. [51]. For Southern blot analysis, rice genomic DNA was isolated from young leaves in 96 tube-tacks (Qiagen) by dry-grinding using a Mixer Mill MM300 (Retch, Germany) with 4 mm stainless steel beads followed by DNA extraction according to Pereira and Aarts [52]. Ten μg of DNA per sample was digested with *Hin*dIII (only one cut site in the T-DNA region), fractionated on a 0.8% agarose gel run in TAE and transferred onto Hybond N^+^ membranes (Amersham) under alkaline conditions. The hygromycin phosphotransferase II (*hptII*) gene (1 Kb) was excised from vector pC1300intB-35SnosBK (GenBank accession AY560326) as *Xho*I fragment. Hybridizations were performed with ^32^P-labelled *hptII*-probe at 65°C in hybridization mixture (10% dextran sulphate, 1 M NaCl, 1% SDS, 100 μg/mL of denatured salmon sperm DNA). The membranes were washed once in 2X SSC and 1% SDS at 65°C for 30 minutes and once in 2X SSC and 0.1% SDS at 65°C for 30 minutes. For northern blot analysis, 20 μg of total RNA per sample was electrophoresed in formaldehyde agarose gel and transferred to Hybond-N^+^ membrane. Baked blots were (pre)-hybridized in 1 M NaCl, 1% SDS, 10% dextrane sulfate and 50 μg/mL denatured herring sperm DNA at 65°C, washed with 0.1 XSSPE and 0.5% SDS at 42°C and autoradiographed. Probes were labeled by random priming with ^32^P-dCTP. Equal loading of RNA samples was verified on the basis of ethidium bromide staining of ribosomal RNA bands.

### GA treatment of plants

To evaluate the response of sheathed panicle to exogenous GA, transformed lines overexpressing *Oshox14* lines with strong phenotype were sprayed with 20 μM GA_3_ (Gibberellic acid) at the end of panicle differentiation. For each independent line, five transformed plants were treated.

### Histochemical localization of GUS activity

Plant materials were vacuum infiltrated for 20 min in GUS staining solution containing 100 mM phosphate buffer pH 7.7, 2 mM 5-bromo-4-chloro-3-indolyl-β-D-glucuronic acid (X-Gluc; Biosynth AG), 0.5 mM potassium ferricyanide, 10 mM EDTA and 0.1% Triton X-100 and incubated at 37°C for 1 h to overnight, depending on staining intensity. The samples were cleared by several changes of 70% (v/v) ethanol and stored at 4°C.

For sectioning, the samples were dehydrated in a graded ethanol series from 70% to 100% and embedded in Technovit 7100 resin (Kurzer, Wehrheim, Germany), polymerized at 37°C, and cut into 3–5 μm sections that were stained with toluidine blue. The samples were viewed using a Leica MZ12 stereo microscope or a Leitz Diaplan microscope with bright-field optics settings, and images were acquired with a Sony 3CCD Digital Photo Camera DKC-5000. Greenhouse-grown plants were photographed with a Canon EOS 350D camera.

## Results

### Phylogenetic analysis of *Oshox12* and *Oshox14*

Previous work has shown that the barley *Vrs1* gene suppresses the development of lateral spikelets and that loss of function in *Vrs1* lines results in complete conversion of two-rowed barley into six-rowed barley [34, 36]. BLAST searches of the rice (http://rice.plantbiology.msu.edu/analyses_search_blast.shtml) with Vrs1, found that the rice HD-Zip I family Oshox14 and Oshox12 proteins had the highest similarity. Oshox14 is the closest homologue to Vrs1 based on the sequence comparison, but Oshox14 is not highly expressed in panicles compared to Oshox12 [12]. Thus, Oshox14 may be closer to Vrs1 in function than Oshox12 is. In *Arabidopsis*, the HD-Zip I members Athb-53, Athb-21 and Athb-40 are closest to Vrs1 and HVhox2 [36]. Furthermore, previous studies have shown that rice Oshox12, Oshox14 and *Arabidopsis* Athb-53, Athb-21, Athb-40 are all in the so-called δ clade, which is characterized by a unique intron between the fourth and fifth leucine of the zipper region (the so-called L4-L5 group) whereas all other family I HD-Zips in rice have the intron between L5 and L6 [12].

To further determine the evolutionary distances among these HD-Zip I proteins and Vrs1, a systematic phylogenetic analysis of the HD-Zip I proteins isolated from *Arabidopsis*, barley and rice was performed. This phylogenetic analysis confirmed that Oshox12, Oshox14 and Vrs1 were in the same clade (S1A Fig). Alignment of the entire amino acid sequence showed that rice Oshox14 shared the maximum amino acid sequence similarity with Vrs1, and the degree of full length protein sequence identity to Vrs1 reached 63.27% (S1B Fig); in contrast, Oshox12 shared 43.09% identity with Vrs1 (S1B Fig). These results suggest that *Oshox12* and *Oshox14* might have the same function as *Vrs1*.

The cDNA sequences of *Oshox12* and *Oshox14* are 1,170 bp and 1,173 bp in length, encoding proteins of 239 and 240 amino acids, respectively. The *Oshox12* cDNA sequence includes an ORF of 720 bp with a 5’-UTR of 213 bp and a 3’-UTR of 238 bp, while the *Oshox14* cDNA has an ORF of 723 bp with a 5’-UTR of 206 bp and a 3’-UTR of 245 bp (S1B Fig). Oshox12 and Oshox14 both carry putative nuclear localization signal (NLS) sequences according to the software tools Nucpred and PredictNLS.

#### Interaction of Oshox12 and Oshox14 with the AH1 (CAAT(A/T)ATTG) sequence in yeast

**Fig 1:**
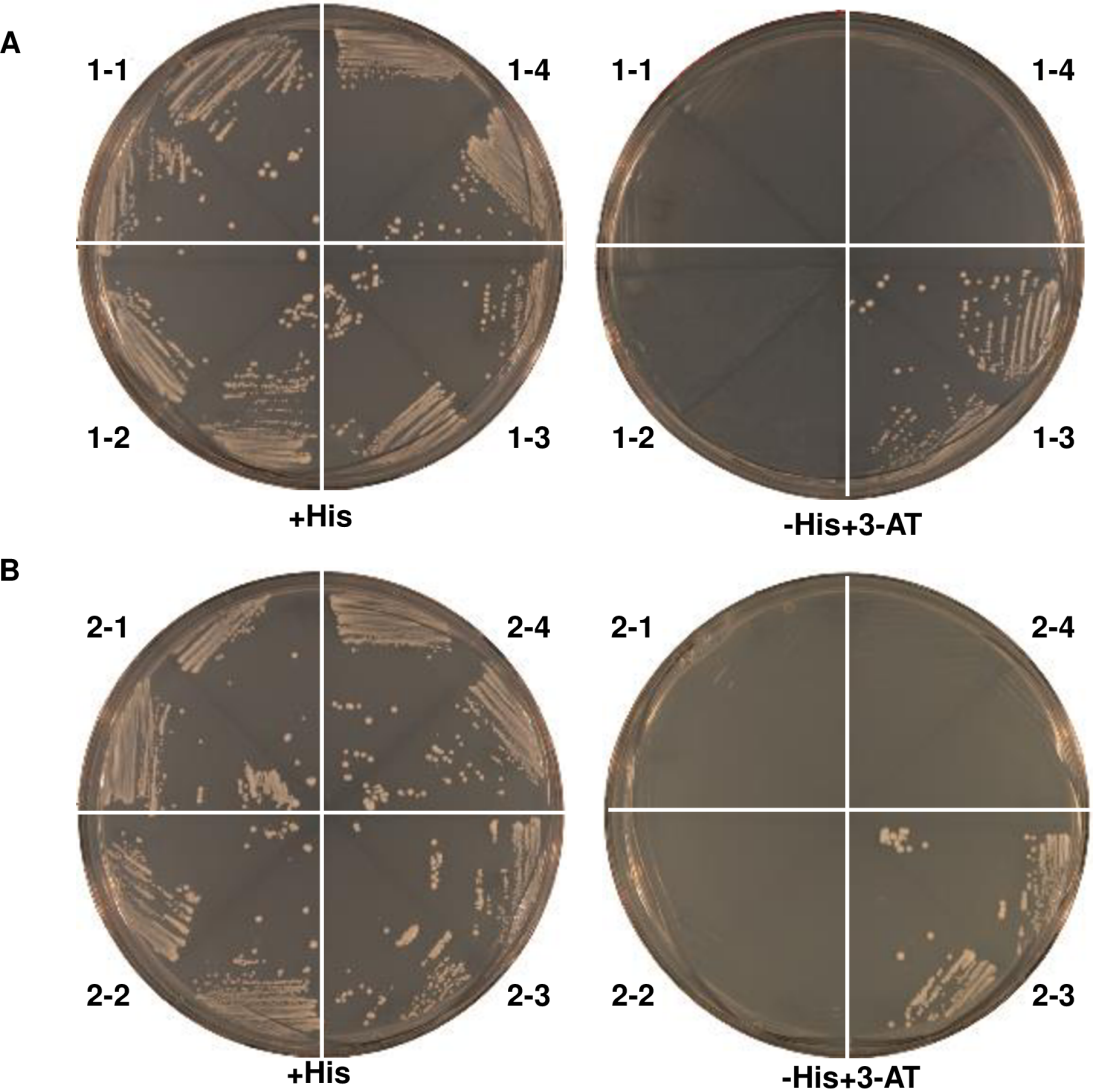
Yeast one-hybrid assays with *Oshox12* and *Oshox14* expression constructs. **A to D,** pRED-ATGb-Oshox12 (A and B) or pRED-ATGa-Oshox14 (C and D) in different yeast strains streaked on medium containing histidine (A and C), or medium without histidine and supplemented with 10 mM 3-AT (B and D). pRED-ATGb-Oshox12 and pRED-ATGa-Oshox14 were transformed into yeast strains YM4271::4AH1 (1–3, 2–3) and YM4271::4AH2 (1–4, 2–4), respectively. The pRED-ATGb and pRED-ATGa empty vectors were used as negative control and tested in YM4271::4AH1 (1–1, 2–1) and YM4271::4AH2 (1–2, 2–2). Growth of colonies on plates without histidine indicates specific activation of the expression of the 4AH1:HIS3 constructs by the pRED-ATGb-Oshox12 or pRED-ATGa-Oshox14 expression constructs.

Previous studies have demonstrated that HD-Zip family I members can bind to the 9 bp pseudopalindromic sequences AH1 (CAAT(A/T)ATTG) and AH2 (CAAT(C/G)ATTG) [43, 53]. To confirm affinities of Oshox12 and Oshox14, we studied the binding of Oshox12 and Oshox14 using yeast one-hybrid system. For this experiment, yeast strains containing a chromosomally integrated *HIS3* reporter gene preceded by upstream AH1 (construct 4AH1-HIS3) or AH2 (construct 4AH2-HIS3) tetramers were used. The results showed that the 4AH1-HIS3 yeast strain transformed with either pRED-ATGb-Oshox12 or pRED-ATGa-Oshox14 grew well on a medium lacking histidine but containing up to 10 mM 3-AT (Fig 1A and 1B), whereas no growth was observed in yeast strains with the 4AH2-HIS3 reporter or with the empty pRED-ATGb expression vector. Our results indicate that both Oshox12 and Oshox14 are able to bind the AH1 sequence, but not AH2 in yeast.

#### Interaction of Oshox12 and Oshox14 with the AH1 (CAAT(A/T)ATTG) sequence in rice

**Fig 2:**
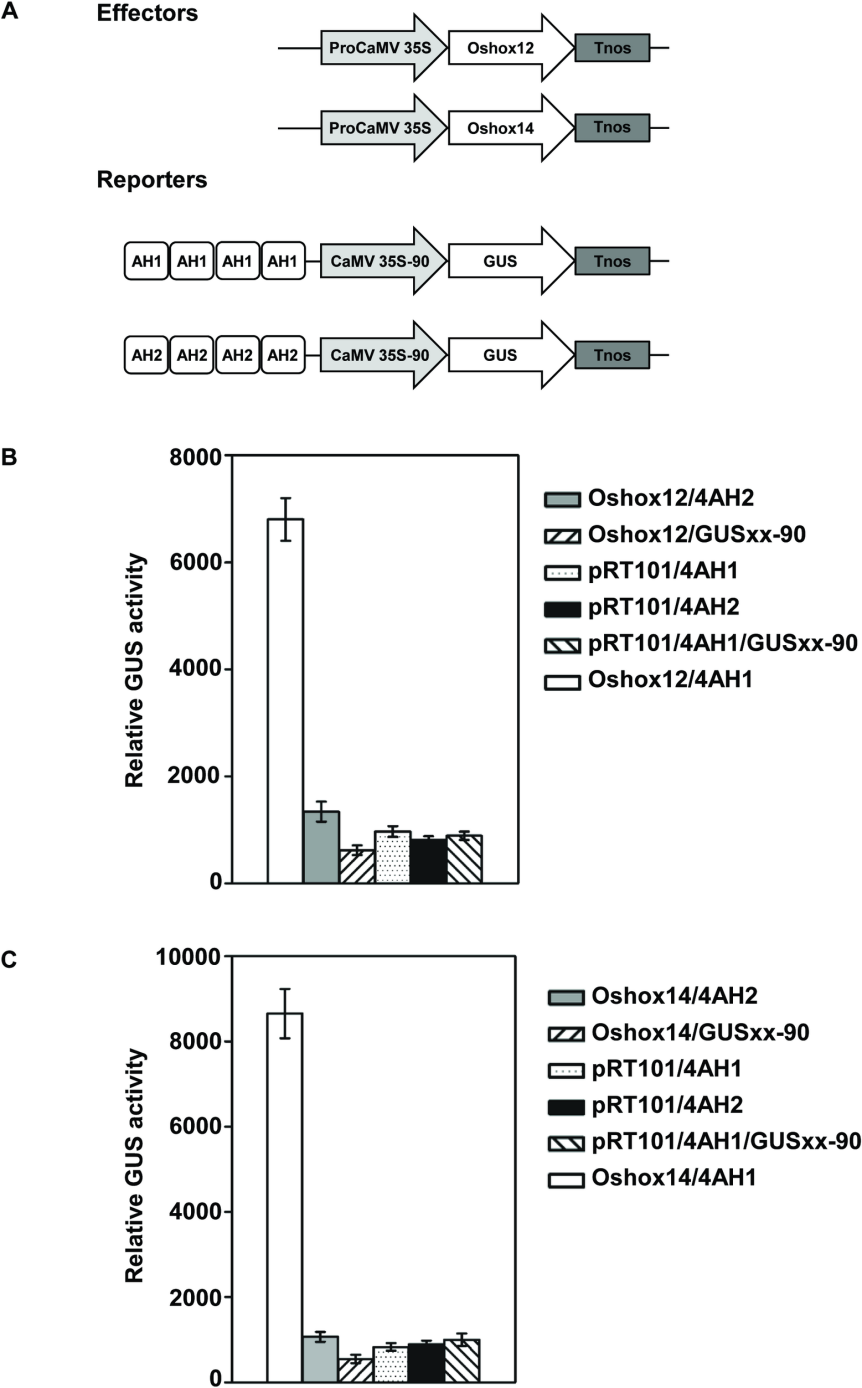
Interactions of Oshox12 and Oshox14 with the HD-Zip binding site AH1 (CAAT(A/T)ATTG) in a transient expression system using rice protoplasts. (A) Schematic overview of the effector and reporter constructs used for transactivation analysis in rice protoplasts. (B-C) Transient expression of *Oshox12* (B) and *Oshox14* (C) was driven by the CaMV 35S promoter and the Oshox12-OX and Oshox14-OX constructs were co-transformed with the reporter constructs GUSXX-4AH1 and GUSXX-4AH2. The empty vectors pRT101 and GUSXX-90 were used as negative controls.

To further confirm binding of the Oshox12 and Oshox14 proteins to the AH1 sequence, transient expression assays were carried out with effector and reporter plasmids in rice protoplasts. Two reporter plasmids, 4AH1-90-GUS and 4AH2-90-GUS, in which the AH1 and AH2 tetramers were fused to a CaMV-90 CaMV 35S minimal promoter were used [54]. The constructs Pro35S-Oshox12 and Pro35S-Oshox14, which contained Oshox12 and Oshox14 expressed under control of the CaMV 35S promoter, were used as effectors (Fig 2A). The GUS expression in protoplasts indicates that Oshox12 and Oshox14 are capable of activating transcription of the reporter gene when the upstream HD-Zip binding site AH1 is present, but cannot activate transcription of the reporter gene when upstream HD-Zip binding site AH2 is present (Fig 2B and 2C). These results show that Oshox12 and Oshox14 can bind specifically to the AH1 (CAAT(A/T)ATTG) DNA sequence and activate reporter gene expression in rice protoplasts, which is consistent with the result obtained in the yeast experiments and is also in line with results obtained for other HD-Zip I and II proteins in gel shifts and yeast experiments with AH1 and AH2 [42, 43].

#### Subcellular localization of Oshox12 and Oshox14 and expression pattern of ProOshox12-GUS and ProOshox14-GUS in rice

**Fig 3:**
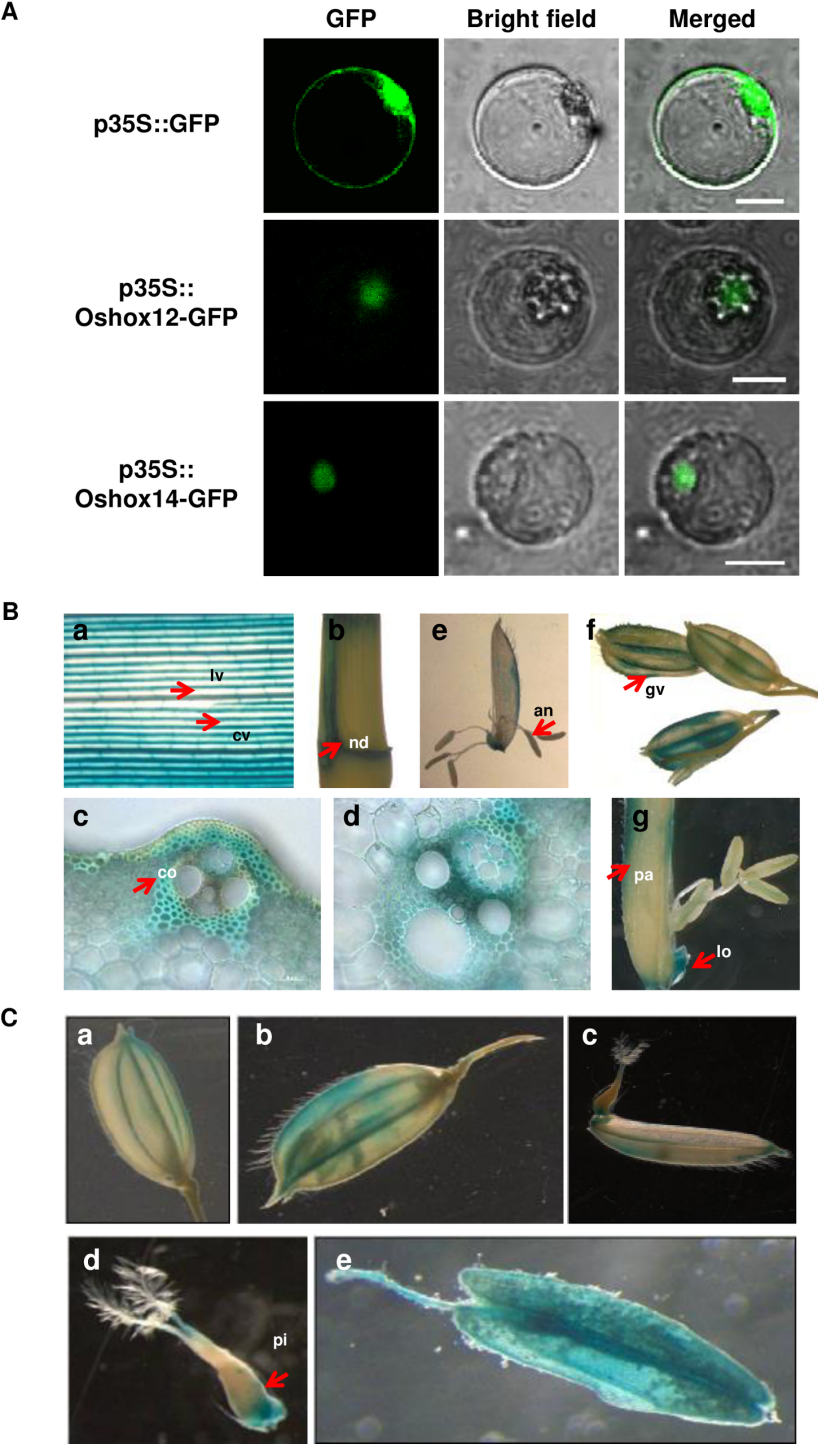
Subcellular localization analysis of Oshox12 and Oshox14 and expression patterns of ProOshox12-GUS and ProOshox14-GUS. (A) Localization analysis of Oshox12-GFP and Oshox14-GFP fusion proteins in rice protoplasts. The scale bar represent 2 μm. (B) Tissue expression pattern of ProOshox12 examined by histochemical GUS staining. Flag leaves (a); stem (b); transverse stem sections of transgenic lines (c, d); anther (e); glume (f); palea and lodicules (g). (C) Tissue expression pattern of ProOshox14 examined by histochemical GUS staining. Mature spikelets before anthesis (a); mature spikelet after anthesis (b); open flower with pistil (c, d), and anther (e). cv, commissural vein. lv, longitudinal vein. nd, node. gv, glume vascular. co, collenchyma. pa, palea. lo, lodicules. pi, pistil.

To study the subcellular localization of Oshox12 and Oshox14, full length *Oshox12* and *Oshox14* clones were fused in frame to GFP, expressed from the CaMV 35S promoter and observed in transiently transformed rice protoplasts. As shown in Fig 3A, in the control vector, GFP signals were observed in both the cytosol and the nucleus. In contrast, we observed that the *Oshox12-GFP* and *Oshox14-GFP* signals were located exclusively in the nucleus, suggesting that both Oshox12 and Oshox14 are nuclear-localized proteins (Fig 3A).

The expression patterns of *Oshox12* and *Oshox14* were further studied using a promoter-GUS fusion construct. In total 20 and 33 independent transgenic Nipponbare lines were made that expressed the constructs ProOshox12-GUS and ProOshox14-GUS, respectively. *GUS* reporter gene activity was detected in seedlings and in tissues of mature plants. The X-Gluc staining showed that *Oshox12* is expressed in nodes and young leaves and in the vegetative growth stage, highly expressed in glume, anther, palea and lodicules in mature plants (Fig 3B). Previously, RT-PCR results showed that *Oshox12* was predominantly expressed in panicles at 10 and 15 DAF [12]. The GUS staining result is consistent with the *Oshox12* expression profile deducted from the Rice Genome Annotation Project (RGAP, http://rice.plantbiology.msu.edu/index.shtml) Database (S2A Fig) and a recent work by Gao et al. [55]. ProOshox14-GUS was mainly expressed in the reproductive organs, such as anther and pistil (Fig 3C), which is also consistent with the results from the RGAP Database (S2A Fig). Although the RT-PCR results implicated that the highest level of *Oshox14* expression was found in the stem rather than in the other detected organs, no expression of *Oshox14* in leaf sheath [12]. In addition, the available microarray-based expression profile for different development stages suggest that both *Oshox12* and *Oshox14* are highly expressed in calli, hull and panicle, with low expression in the radicle and root (S2B Fig) [56].

#### Phenotypes of transgenic rice plants overexpressing *Oshox12* and *Oshox14*

**Fig 4:**
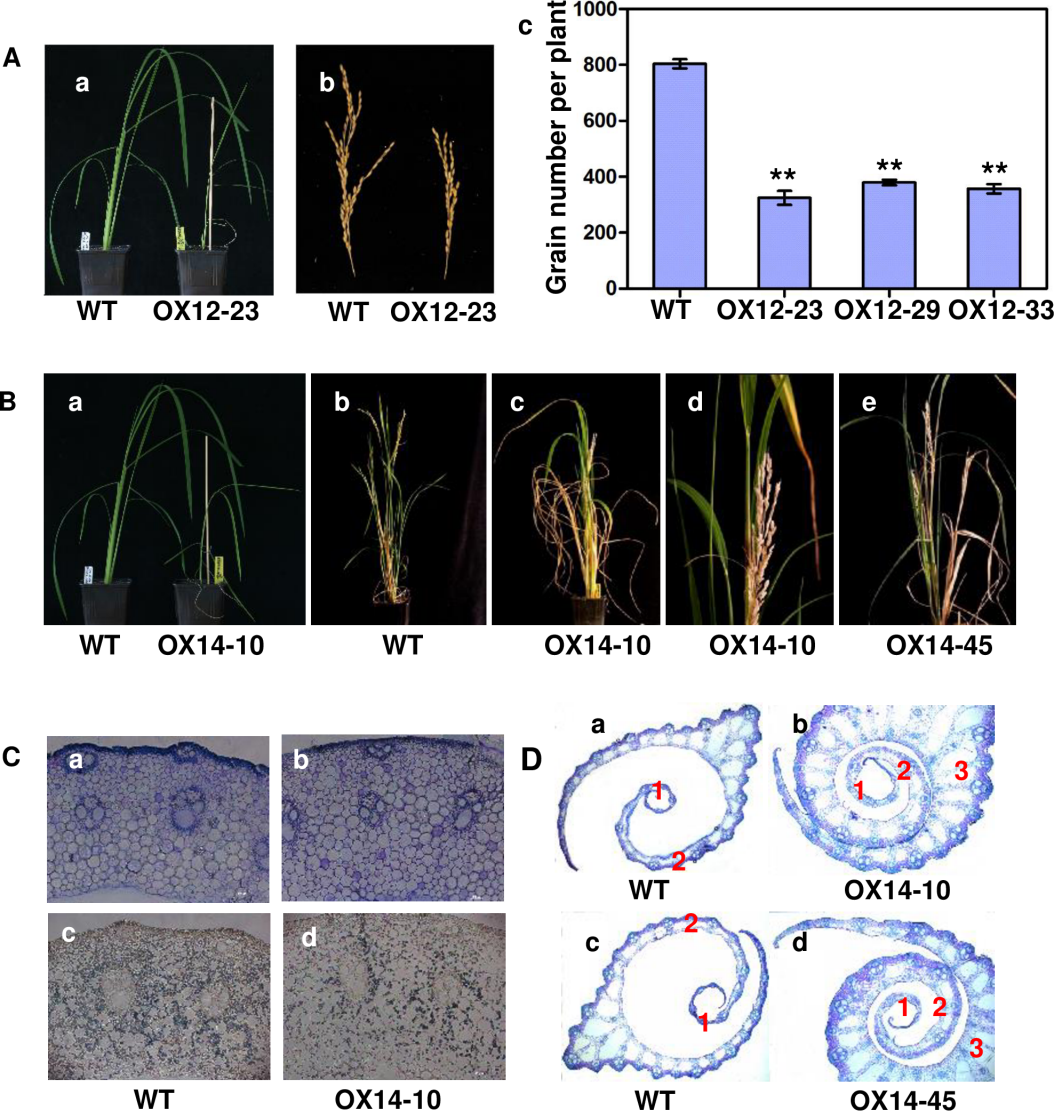
Phenotypes of Pro35S-Oshox12 and Pro35S-Oshox14 plants. (A) Phenotypes of wild type Zhonghua 11 and Pro35S-Oshox12 overexpression in seedling (a) and panicle stages (b). Grain number of Pro35S-Oshox12 overexpression plants compared with that of the wild type control (c). (B) Phenotype of *Oshox14* overexpression lines. Pro35S-Oshox14 overexpression lines and wild type at the seeding stage (a) and panicles (b, c, e). Panicle exsertion in c after GA_3_ treatment (d). (C) Transverse sections of the stems from wild type Zhonghua 11 and Pro35S-Oshox14 overexpression lines. Toluidine blue staining (a, b) and Lugol staining (c, d) respectively of transversal stem sections. (D) Transverse sections of the leaf sheath from wild type Zhonghua 11 (a, c) and two Pro35S-Oshox14 overexpression lines (b, d). The numerals 1–4 indicate the number of turns the leaf sheath in wild type and Pro35S-Oshox14 overexpression lines.

**Fig 5:**
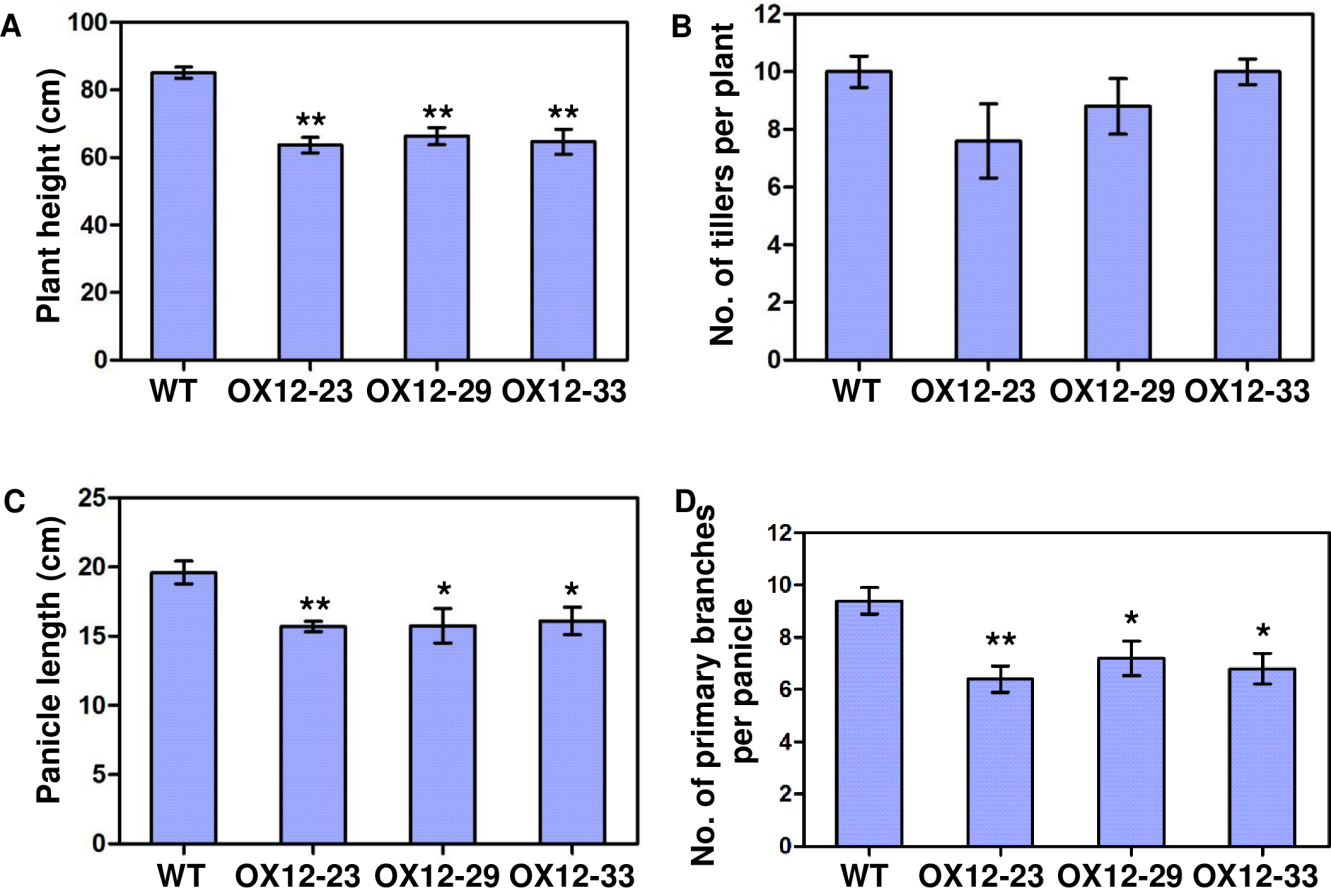
Phenotype of *Oshox12* overexpression transgenic lines at the mature stage. (A) Plant height. (B) Number of tillers per plant. (C) Panicle length. (D) Number of primary branches per panicle. Bars represent standard errors. Data are the average of ten replicates (ten plants). Asterisks indicate significance at * *P*<0.05 and ** *P*<0.01 (Student’s *t*-test). WT, wild type (Zhonghua 11); OX12-23, OX12-29 and OX12-33 are three independent *Oshox12* overexpression lines.

*Oshox12* was further investigated by gain-of-function studies. For this, thirty-one independent T_0_ lines were obtained and overexpression of *Oshox12* was confirmed by northern blot analysis (S3A Fig). Further Southern blot analysis showed that 16 lines were single-copy (S3B Fig). Three independent transgenic lines (OX12-23, OX12-29 and OX12-33), with high expression levels and obvious phenotypic differences were selected for further phenotyping. We observed that overexpression of *Oshox12* induces a semi-dwarf phenotype accompanied by low fertility (Fig 4A). Although both wild type and Oshox12-OX lines had five nodes at maturity, plant height of *Oshox12* transgenic plants was reduced because of the shortened uppermost internode (data not shown). The average plant heights of the three Oshox12-OX lines were 63.7 cm, 66.34 cm and 64.7 cm, respectively, whereas that of wild type plants reached 85.1 cm on average (Fig 5A). Thus, the average plant height in the three Oshox12-OX lines was decreased by 23% (P<0.01). Furthermore, the Oshox12-OX lines displayed a decrease in tiller number (though this effect was not significant) (Fig 5B).

*Oshox12* is predominantly expressed in the panicle suggesting that it has a function in the development of this tissue [12]. An examination of the panicle architecture in the Oshox12-OX lines revealed significant decreases in panicle axis length and primary branch number (Fig 5C and 5D). In the transgenic plants, the main panicle length was reduced by 20% from an average of 19.6 to 15.6 cm (*P* < 0.01, n = 10) (Fig 5C). The number of primary branches per main panicle (Fig 5D) was also determined. On average, panicles from lines OX12-23, OX12-29 and OX12-33 had 6.4, 7.2 and 6.8 primary branches, respectively, while the wild type panicles had 9.6 primary branches, representing a significant reduction in the Oshox12-OX lines (Fig 5D). In addition, we found the grain number to be reduced in the Oshox12-OX lines (Fig 4A panels b-c). On average, the grain numbers from lines OX12-23, OX12-29 and OX12-33 were 324.6, 379.6 and 357, respectively, while the wild type had 803.8 grains per plant; this reduction was also significant. Taken together, these data indicate that *Oshox12* might be involved in panicle development.

Our next step was to examine whether Oshox14 functions as a developmental regulator and to determine whether it shows functional similarities to Vrs1, for which purpose we made transgenic rice plants overexpressing *Oshox14*. Thirty-four independent T_0_ lines were obtained and over-expression of *Oshox14* was confirmed by northern blot analysis (S4A Fig). Southern blot analysis showed that four lines were single-copy (S4B Fig). Three primary transformants with high *Oshox14* expression levels of Oshox14, containing the sense gene construct (lines OX14-9, OX14-10, OX14-45) were found to be severely retarded in growth at the seeding stage (Fig 4B panel a) and showed difficulties with panicle exsertion through stem and leaf sheath at the mature stage (Fig 4B panels c, e). The plants with the strongest phenotypes showed fully sheathed panicles. To clarify whether this defect was accompanied by abnormalities in leaf sheath development or internode elongation, we performed an anatomical study of sections from the first internodes and leaf sheath. The results showed that no difference between the first internodes at anatomical level (Fig 4C panels a, b); however, the Lugol staining experiment showed decreased starch content in stems of the Oshox14-OX plants (Fig 4C panels c, d). Further histological sectioning showed that additional differences in the structures of the leaf sheath. In general, the *Oshox14* overexpressing lines have more turns of the flag leaf sheath than that of the wild type (Fig 4D). The severity of the phenotype in these transgenic plants was correlated with the expression levels found in northern blotting (S4A Fig). It is known that the leaf sheath from rice elongates rapidly in response to treatment with GA [57]. Thus, we treated *Oshox14* overexpressing lines with the strong phenotypes with 20 μM GA_3_ at the end of panicle differentiation, which led to the panicle being exserted from the culm and the flag leaf (Fig 4B panel d).

**Fig 6:**
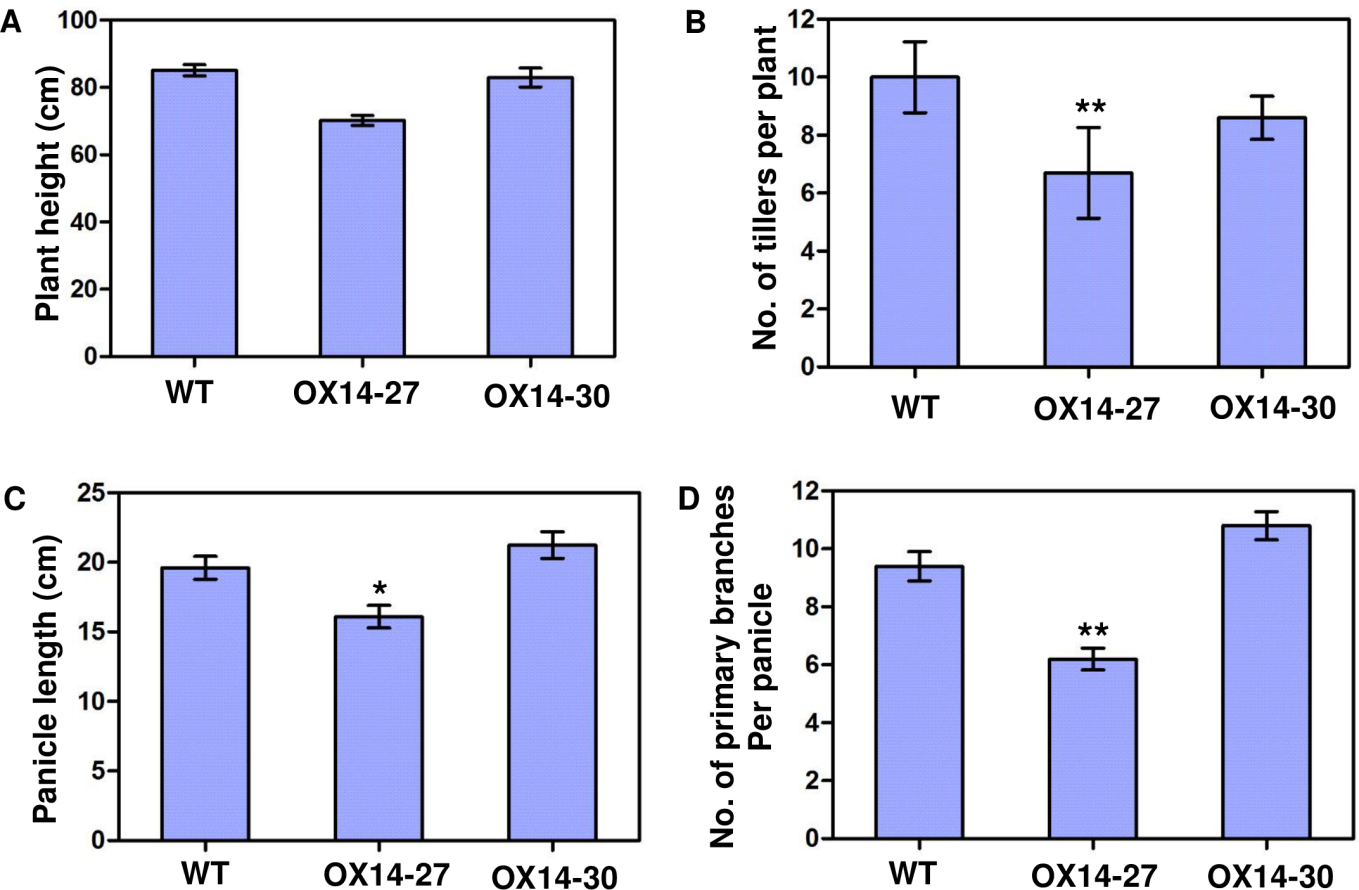
Phenotype of *Oshox14* overexpression lines at the mature stage. (A) Plant height. (B) Number of tillers per plant. (C) Panicle length. (D) Number of primary branches per panicle. Bars represent standard errors. Data are the average of ten replicates (ten plants). Asterisks indicate significance at * *P*<0.05 and ** *P*<0.01 (Student’s *t*-test). WT, wild type (Zhonghua 11); OX14-27, a low-overexpression line of *Oshox14;* OX14-30, a zero overexpression line of *Oshox14*.

Due to the phenotypic abnormalities in the lines with weaker phenotypes, we could obtain only a small number of T_2_ seeds for further study, which included phenotyping for plant height, tiller number, main panicle length and numbers of primary branches per panicle (Fig 6). Though line OX14-27 displayed only a non-significant decrease in plant height (Fig 6A), the tiller number, main panicle length and numbers of primary branches per panicle, were significantly different than those of the wild type (Fig 6B–6D). Examination of the panicle architecture in the zero expression line OX14-30 showed no difference from that of the wild type (Fig 6). This result may be explained by the weak overexpression of the *Oshox14* construct in OX14-27.

## Discussion

The HD-Zip TF family is one of the largest super-families of homeobox genes in plants [6–9, 12] and an increasing amount of knowledge is being acquired about their functions in rice [7, 27, 28, 54, 58]. In barley, the HD-Zip I member *Vrs1* is important in determining grain yield [34, 36] via an effect on inflorescence architecture. The architecture of the inflorescence plays a key role in the determination of grain yield, but our understanding of the genetic control of this complex trait is still limited [59]. In rice, the HD-Zip I genes, *Oshox12* and *Oshox14* are close homologues of *Vrs*1 [12, 36]. Based on an overexpression analysis of these two genes, we propose a function of *Oshox12* and *Oshox14* in panicle and sheath development.

The model plants *Arabidopsis* and rice have 17 and 14 HD-Zip I genes, respectively [11, 12]. Rice *Oshox12* and *Oshox14* and *Arabidopsis Athb-21*, *Athb-40* and *Athb-53*, belong to a relative small subfamily characterized by an intron between the fourth and fifth leucine of the zipper region (originally called the δ clade or L4–L5 group) whereas many other HD-Zip I genes have an intron between the fifth and sixth leucine of the zipper region [11, 12]. The transcript levels of *Arabidopsis Athb-21*, *Athb-40* and *Athb-53* are upregulated upon exposure to ABA and salinity stress [14] and these genes are thought to be involved in ovule development [60]. During root development, *Athb-53* also plays an important role in auxin/cytokinin signaling [61]. Based on phylogeny, a set of 178 HD-Zip I proteins from different plant species was divided into six groups (I to VI) [62]. Based on this analysis, the δ clade members Oshox12 and Oshox14 [12] were included in group VI [62]. Furthermore, this analysis revealed a set of 20 conserved motifs in the amino-terminal (NTR) and carboxy-terminal regions (CTR). Group VI proteins, including Oshox12 and Oshox14 share in common that they have a so-called motif 10 in the NTR which is also unique to this group but for the moment a precise function is yet unclear although some group VI proteins have a nuclear localization signal in motif 10 [62]. In addition, Oshox12 and Oshox14 have three and six putative phosphorylation sites (Ser, Thr, Tyr), respectively in the CTR, but no sumoylation site was found. Both Oshox12 and Oshox14 possess the so-called AHA (Aromatic, large Hydrophobic, Acidic context) motif in the CTR, which is responsible for transcriptional activation. In addition, both TFs contained a high frequency of aromatic amino acid phenylalanine (Phe) in the CTR but a precise function for this phenomenon is unclear yet [62].

Consistent with the known function of TFs, the GFP-tagged fusion constructs indicated that Oshox12 and Oshox14 are both nuclear-localized proteins. A similar result for Oshox12 was also reported elsewhere [55]. In general, HD-Zip I family members bind *in vitro* and *in vivo* to the 9-bp pseudopalindromic *cis*-element, AH1 (CAAT(A/T)ATTG) and AH2 (CAAT(C/G)ATTG [42, 43, 52, 54]. Our yeast one-hybrid experiment suggests that Oshox12 and Oshox14 specifically bind to the proposed AH1 sequence. It is obvious that these proteins can activate reporter gene expression by an intrinsic activation domain which was also observed for other family I proteins from rice [27, 43]. Oshox12 and Oshox14 as transcriptional activators were further confirmed by transient assays in rice protoplasts using the *GUS* reporter gene. HD-Zip TFs generally form homodimers or heterodimers to regulate downstream gene expression [17, 42]. Oshox12 was shown that it can form homodimers as well as heterodimers with Oshox14 in a bimolecular fluorescence complementation (BiFC) system [55]. Interestingly, both Oshox12 and Oshox14 can also interact with *ELONGATED UPPERMOST INTERNODE1* (*EUI1*) in yeast one-hybrid and electrophoretic mobility shift (EMSA) assays, and the *EUI1* gene contains a similar AH1 (CAAT(A/T)ATTG) sequence element in its promoter region [55]. Taken together, the results of the yeast and protoplast experiments support the functions of Oshox12 and Oshox14 as transcriptional activators, which is characteristic of HD-Zip I family TFs [43, 44, 63].

Several sets of transcriptome data have shown that *Oshox12* and *Oshox14* are highly expressed in the panicle [12, 55]. Like *Oshox12*, *Oshox14* is mainly expressed in the panicle, even though its expression level in the panicle is less than that of *Oshox12* [12]. In barley, the *Oshox12* and *Oshox14* homologue, *Vrs1*, is involved in determining the number of rows of spikelets by suppressing the development of lateral rows [34]. Base on the microarray data, the expression patterns and our phylogenetic analysis, we suggest that *Oshox12* and *Oshox14* might be involved in panicle development, which is further supported by our promoter-GUS expression analysis. This experiment revealed that *Oshox12* displayed a tissue specific pattern with the highest expression in glume, anther, palea and lodicules. Our data suggest that *Oshox12* function is necessary in different tissues and that this gene may be involved in panicle development. According to the GUS analysis, the promoter of *Oshox14* was also mainly expressed in reproductive organs, such as anther and pistil.

Defects in the elongation of the uppermost internode lead to panicle enclosure and thus greatly reduce seed production by blocking normal pollination [64]. Our previous work has shown that *Oshox12* and *Oshox14* are highly expressed in panicles, suggesting a role in panicle development [12]. Consistently, our findings here demonstrate that overexpression of *Oshox12* results in reduced length of the panicle axis, reduction of primary branch number and a consequent decrease in grain yield (Fig 4A and Fig 5). At the heading stage, the *Oshox12* overexpression lines exhibited a shortened uppermost internode thereby reducing plant height (Fig 5A). Independently of our work, it was recently demonstrated that *Oshox12* regulates panicle exsertion in rice by directly modulating the expression of *EUI1*, which encodes a cytochrome P450 monooxygenase *CYP714D1* that deactivates bioactive GAs and plays a crucial role in panicle exsertion in rice. Panicle exsertion principally depends on the elongation of the uppermost internode [55]. In rice, there are six groups of internode elongation mutants, which are classified based on the elongation pattern of the upper internodes [65]. In the‘sh’ type, the uppermost internode shows no elongation with the panicle enveloped in the leaf sheath, which results in a sheathed panicle. The rice leaf sheath is an important part of the plant where considerable critical metabolic and regulatory activities occur, and these processes eventually control rice height and robustness. Several mutants with sheathed panicle phenotypes have been identified, including *shp1-5*, *dsp1*, *sui1-1* and *sui1-2* [63]. However, the mechanism underlying sheathed panicles remains unclear. In this study, through the overexpression of *Oshox14*, we found that transgenic plants overexpressing *Oshox14* display sheathed panicles, showing that the overproduction of *Oshox14* also alters panicle development. Microscopic analysis indicates that the cells in the uppermost internode appear the same in the wild type and the *Oshox14* overexpression line, but that the starch content of the transgenic plant stems was decreased. Our experiments with GA treatment showed that the function of *Oshox14* in panicle exsertion may relate to GA signaling. It was reported that *Oshox12* is also involved in regulating panicle exsertion and response to endogenous GA [55]. Thus, in summary, our results strongly suggest that both *Oshox12* and *Oshox14* play important roles in regulating the length of the uppermost internode, probably via GA signaling.

In this study, we demonstrate the roles of *Oshox12* and *Oshox14* in panicle and sheath development. Improving crop productivity by selection for the components of grain yield and for optimal plant architecture has been the key focus of national and international rice breeding programs. However, the detailed molecular mechanisms by which *Oshox12* and *Oshox14* regulate panicle development remain largely unknown, and further genetic analyses of downstream target genes need to be undertaken, including the use of mutant alleles. Elucidation of these downstream events will be one of the keys in understanding the roles of these HD-Zip I TFs and their potential in rice yields improvement.

## Supporting information

S1 Fig Phylogenetic and sequence analysis. (A) Phylogenetic tree showing the predicted relationship of HD-Zip I proteins from rice, Arabidopsis and barley. (B) Sequence alignment of Oshox12, Oshox14 and Vrs1 amino acid sequences.
(TIF)

S2 Fig Expression levels of *Oshox12* and *Oshox14* in different tissues. (A) Expression of *Oshox12* (a) and *Oshox14* (b) in different tissues from the Rice Genome Annotation Project (RGAP, http://rice.plantbiology.msu.edu/index.shtml) Database. (B) Microarray based expression file of *Oshox12* (blue line) and *Oshox14* (purple line) in rice at various developmental stages.
(TIF)

S3 Fig Northern and Southern blotting analyses of Pro35S-Oshox12 transgenic plants. (A) Northern blotting analysis of Pro35S-Oshox12 transgenic plants. Lane 1 and 2 show wild type controls; the results show that lines 7, 9, 11, 14, 22 to 33 (red numbers) are overexpression lines of *Oshox12*. The *Oshox12* probe was derived from λFLC-1-B-Oshox12 digested with *Bam*HI and *Eco*RI. The arrow indicates the size of the Oshox12 mRNA overexpressed in the *Oshox12* overexpression lines. (B) Copy number verification of Pro35S-Oshox12 plants by Southern blotting analysis. The *hptII* gene was used as a probe excised from vector pC1300intB-35SnosBK. The results indicate that all 16 lines were single copy for the *Oshox12* overexpression construct.
(TIF)

S4 Fig Northern and Southern blotting analysis of Pro35S-Oshox14 transgenic plants. (A) Northern blotting analysis of Pro35S-Oshox14 transgenic plants. Lanes 1 and 2 show wild type controls; the result show that lines 9, 10, 25 and 45 (red number) are high overexpression lines of *Oshox14*, while numbers 27, 33 are low overexpression lines of *Oshox14*. The *Oshox14* probe was derived from λFLC-1-B-Oshox14 digested with *Kpn*I. The arrow indicates the size of the *Oshox14* mRNA in the overexpression lines. (B) Copy number verification of Pro35S-Oshox14 transgenic plants by Southern blotting analysis. The *hptII* gene was used as a probe excised from vector pC1300intB-35SnosBK. The results indicate that all four lines were single copy of *Oshox14*.
(TIF)
