# Supplementary figures and images for: Functional analysis of the HD-Zip transcription factor genes Oshox12 and Oshox14 in rice
Source: PLoS One. 2018 Jul 20;13(7):e0199248. doi: 10.1371/journal.pone.0199248 (PMC6054374; doi:10.1371/journal.pone.0199248)

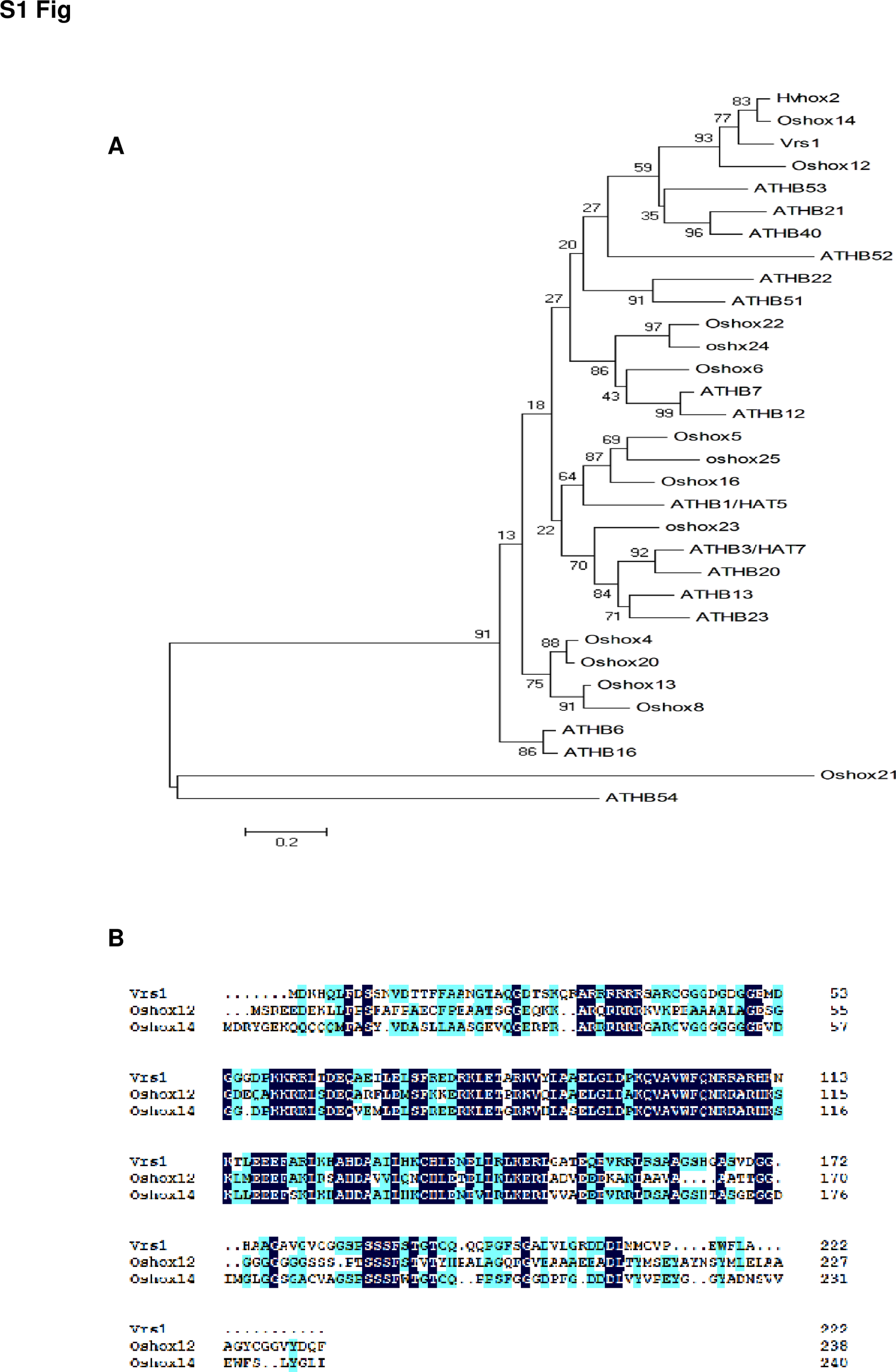

Supplement: S1 Fig — (A) Phylogenetic tree showing the predicted relationship of HD-Zip I proteins from rice, Arabidopsis and barley. (B) Sequence alignment of Oshox12, Oshox14 and Vrs1 amino acid sequences. (TIF) [file pone.0199248.s001.tif]

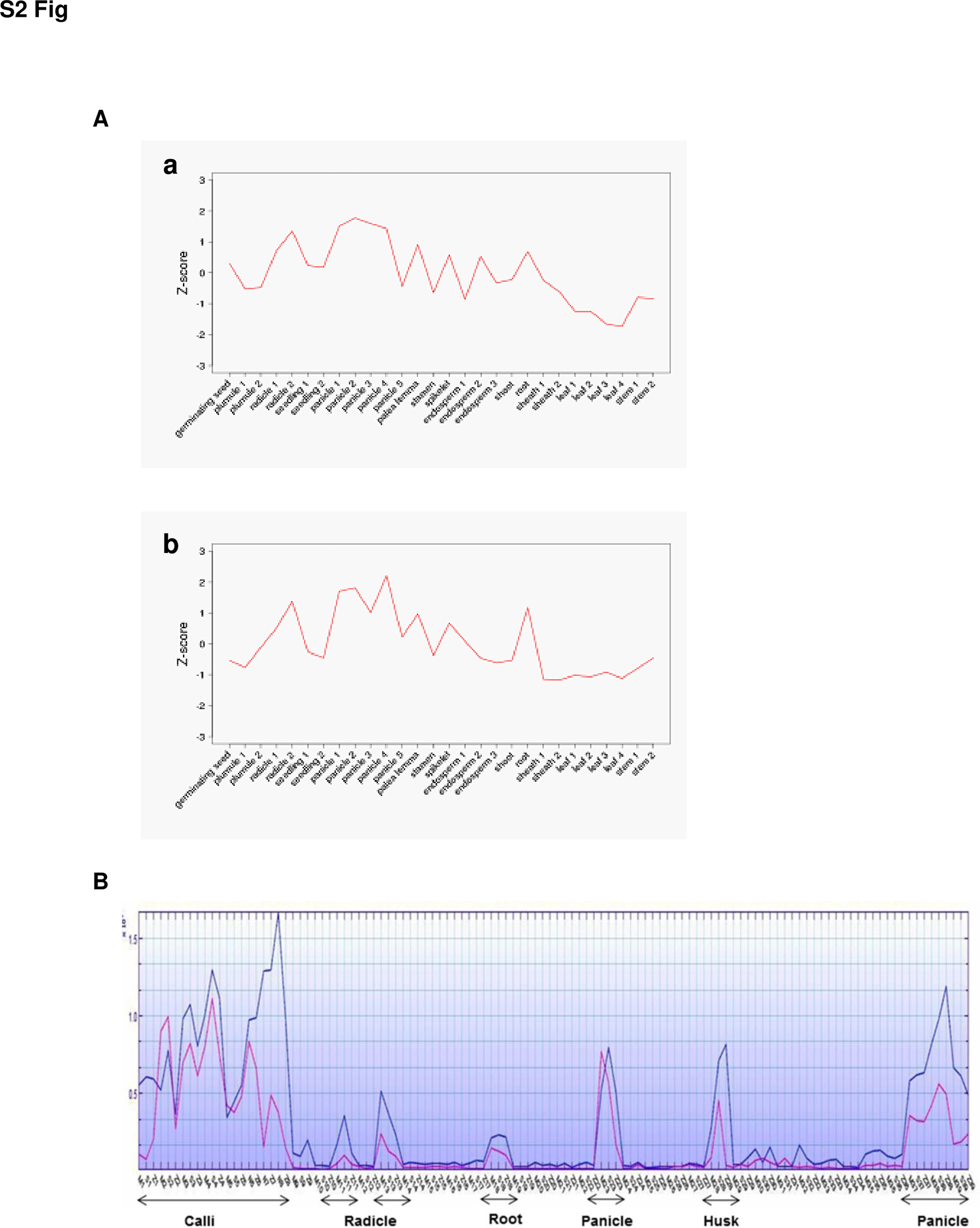

Supplement: S2 Fig — (A) Expression of Oshox12 (a) and Oshox14 (b) in different tissues from the Rice Genome Annotation Project (RGAP, http://rice.plantbiology.msu.edu/index.shtml) Database. (B) Microarray based expression file of Oshox12 (blue line) and Oshox14 (purple line) in rice at various developmental stages. (TIF) [file pone.0199248.s002.tif]

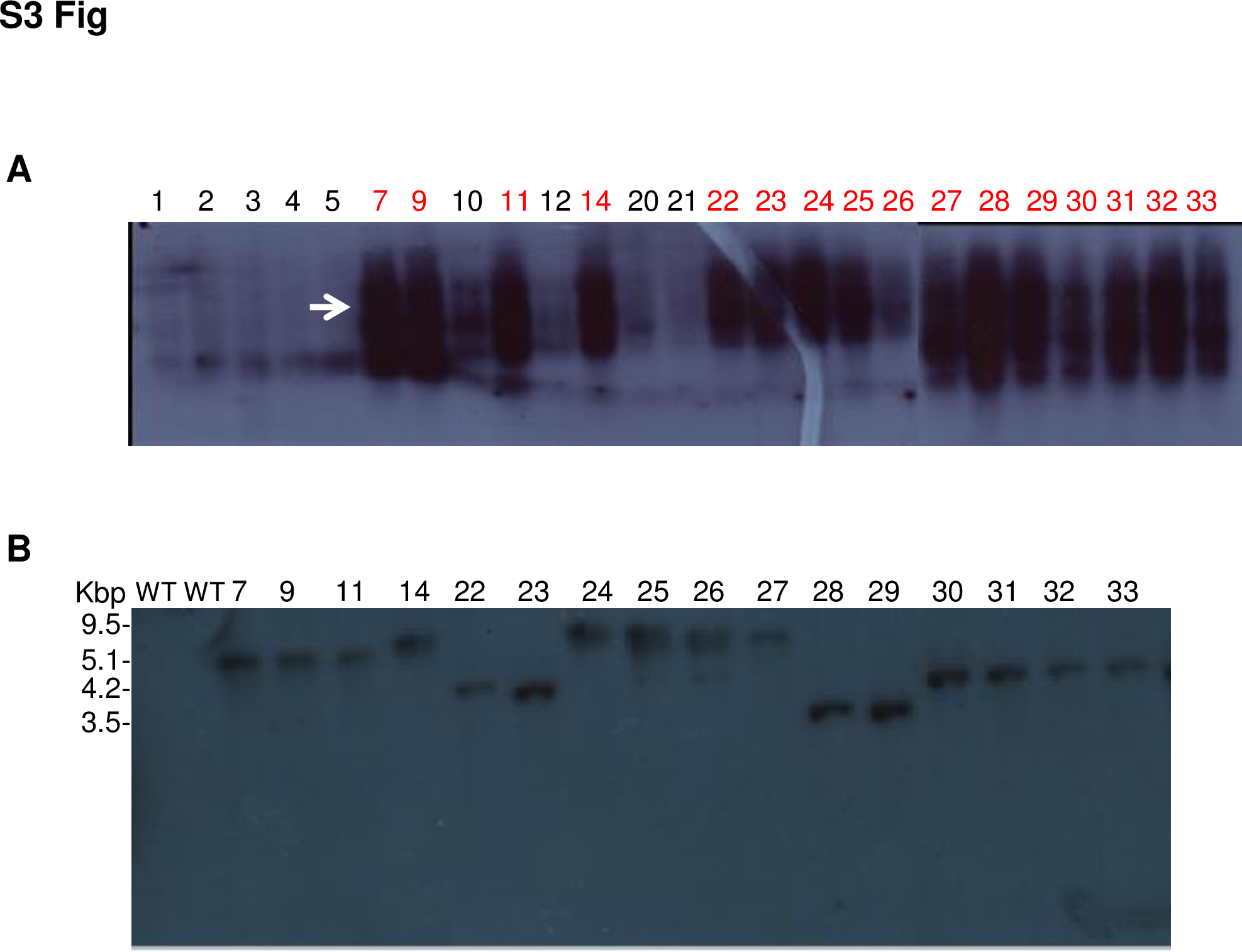

Supplement: S3 Fig — (A) Northern blotting analysis of Pro35S-Oshox12 transgenic plants. Lane 1 and 2 show wild type controls; the results show that lines 7, 9, 11, 14, 22 to 33 (red numbers) are overexpression lines of Oshox12. The Oshox12 probe was derived from λFLC-1-B-Oshox12 digested with BamHI and EcoRI. The arrow indicates the size of the Oshox12 mRNA overexpressed in the Oshox12 overexpression lines. (B) Copy number verification of Pro35S-Oshox12 plants by Southern blotting analysis. The hptII gene was used as a probe excised from vector pC1300intB-35SnosBK. The results indicate that all 16 lines were single copy for the Oshox12 overexpression construct. (TIF) [file pone.0199248.s003.tif]

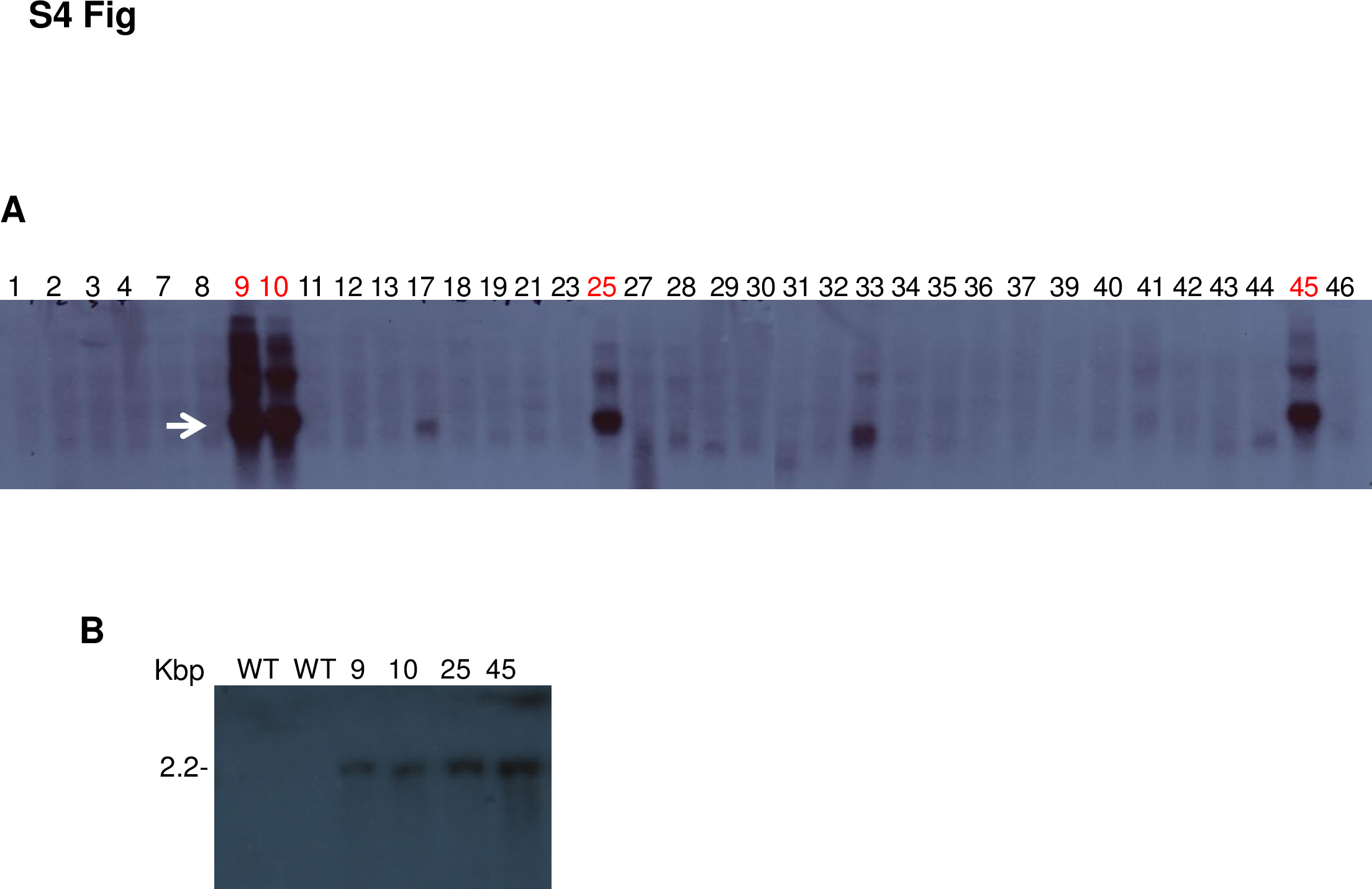

Supplement: S4 Fig — (A) Northern blotting analysis of Pro35S-Oshox14 transgenic plants. Lanes 1 and 2 show wild type controls; the result show that lines 9, 10, 25 and 45 (red number) are high overexpression lines of Oshox14, while numbers 27, 33 are low overexpression lines of Oshox14. The Oshox14 probe was derived from λFLC-1-B-Oshox14 digested with KpnI. The arrow indicates the size of the Oshox14 mRNA in the overexpression lines. (B) Copy number verification of Pro35S-Oshox14 transgenic plants by Southern blotting analysis. The hptII gene was used as a probe excised from vector pC1300intB-35SnosBK. The results indicate that all four lines were single copy of Oshox14. (TIF) [file pone.0199248.s004.tif]
